# What is So Special About Contemporary CG Faces? Semiotics of MetaHumans

**DOI:** 10.1007/s11245-022-09814-0

**Published:** 2022-08-25

**Authors:** Gianmarco Thierry Giuliana

**Affiliations:** Dipartimento di Filosofia e Scienze dell’Educazione, Università degli Studi di Torino, Via Sant′Ottavio 20, 10124 Turin, Italy

**Keywords:** Semotics, Face, Metahuman, Computergraphics

## Abstract

This paper analyses the features of the 2021 software for the creation of ultrarealistic digital characters “MetaHuman Creator” and reflects on the causes of such perceived effect of realism to understand if the faces produced with such software represent an actual novelty from an academic standpoint. Such realism is first of all defined as the result of semio-cognitive processes which trigger interpretative habits specifically related to faces. These habits are then related to the main properties of any realistic face: being face-looking, face-meaning and face-acting. These properties, in turn, are put in relation with our interactions with faces in terms of face detection, face recognition, face reading and face agency. Within this theoretical framework, we relate the characteristics of these artificial faces with such interpretative habits. To do so, we first of all make an examination of the technological features behind both the software and the digital faces it produces. This analysis highlights four main points of interest: the mathematical accuracy, the scanned database, the high level of details and the transformative capacities of these artificial faces. We then relate these characteristics with the cultural and cognitive aspects involved in recognizing and granting meaning to faces. This reveals how metahuman faces differs from previous artificial faces in terms of indexicality, intersubjectivity, informativity and irreducibility. But it also reveals some limits of such effect of reality in terms of intentionality and historical context. This examination consequently brings us to conclude that metahuman faces are qualitatively different from previous artificial faces and, in the light of their potentials and limits, to highlight four main lines of future research based on our findings.

## Introduction

In February 2021, the famous videogame and software company Epic Games released the trailer^1^ for its new software “MetaHuman Creator” (MHC from now on), entirely dedicated to the creation of “High-fidelity digital humans”^2^ called “MetaHumans”. This software was put into early access in April 2021 and received widespread attention for the unprecedented realism of its animated digital faces, to the point of using it for the *Matrix Awakens* (2022) promotional campaign.^3^ MHC is the result of a now thirteen years old R&D project which, in both film and videogames, has one very specific and explicit goal: the creation of realistic digital humans with faces which are able to *lie*.^4^ In the specific case of MHC, however, this R&D project is not only about achieving an aesthetic goal but also about automatizing the creation of realistic digital faces to cut production costs.^5^ In fact, MHC was more conceived as an easy-access tool rather than an invention defining a new boundary for facial photorealism: “We're not claiming to cross the uncanny valley here. We're more targeting The Last of Us/Uncharted kind of look, where it's obviously a digital character but it's a pleasant-looking digital character. […] the goal isn't photorealism […].”.^6^

In light of this, two main interrelated research questions arise:

1. What are the causes of such perceived and attributed realism?
2. Can we consider them as a new type of artificial face?

Since both questions are related to the notion of realism, in the first section of the paper we lay out our semiotic perspective on the issue and justify such theoretical approach in terms of methodological efficiency for the specific case study of MHC. Within this framework, we define four main criteria related to the pragmatic of our interactions with faces and which play a key role in both determining the effect of reality and in recognizing MHC faces as a new type of artificial face.

At that point, the steps to answer our main question are twofold: describing the formal characteristics of metahuman faces and describing the semio-cognitive processes triggered by such faces and resulting in an effect of reality. Section three and four are where the first step is done by making a brief overview of the three main distinguishing technologies at work in MHC and then by exposing the main characteristics of the produced faces. In section five we complete the second step by relating these characteristics with the semiotic and cognitive processes involved in the acknowledgement of realism, thus answering to our research questions. This analysis leads us in Sect. 6 to also discuss some critical factors for which these faces still do not yet fully overcome the issue of the uncanny valley and may still not be truly convincing. Finally, in the conclusions, we respond positively to the recognition of MHC artefacts as a new type of faces and suggest future interdisciplinary studies that could be done on the basis of our results.

## Theory and Methodology

Before any attempt is made to answer our two research questions, we need to avoid any possible linguistic impasse on the question of whether a given thing is realistic. We therefore need a general theoretical perspective on what “realistic” means when applied to an object such as a face.

Commonly, we describe a digital object as realistic when our perceptual experience of it is similar to our experience of that same object existing in the physical world. Digital realism is thus conceived as a comparable phenomenological experience between two objects belonging to two different ontologies (Levy 1997) and responding to a different materiality (Dondero 2020); ideally undermining the distinction between an object and its representation. Realism is therefore an impression of the viewer experienced in front of an artificial object and resulting from a technique.

Without denying the validity of such a view, it presents the problem of almost implying that real objects present themselves to us directly in the physical world while virtual ones are accessible only through more or less transparent mediations that technically determine the impression of realism. For this reason in this paper we will choose a different theoretical perspective that focuses on the face as an object that in order to be recognized both as real and realistic must inevitably be previously interpreted as such. This perspective is a semiotic one, a discipline which does not fully recognize the validity of dualistic distinctions in ontological terms and which is traditionally critical of naturalness (Barthes 1957). As such, the real for semiotics is always a question of construction, mediation, comparison: it is an *effect* determined by an act of interpretation of a given object to which we are exposed. Assuming this perspective, semiotics does not deny the existence of the real, but simply assumes that this real can be known by humans only through fallible inferences made within systems that do not inherently discriminate between the real/false and the represented/artificial. Indeed, the epistemology of semiotics is related to Peirce's philosophy and Saussure's structuralism, two different theories of meaning-making that postulate the same idea: that we do not have sensory-directed, psychologically intuitive, or culturally neutral access to objects and events. Hence, every aspect of our understanding of reality (from explicit attributions of meaning to perception^7^) is always *mediated* and *adjusted* by our cognition and prior knowledge. Which in turn are both dynamically co-determined by socio-historical (Eco 1975) and material (Malafouris 2013) context of the subject. Consequently, both real objects ad their representations can only be grasped by us as signs constituted by a superior/external layer of tangible characteristics and differences (expression level) correlated to a deeper conceptual and rather opaque layer (content level) of meanings which is highly subject to variation.

In its long reflection on the relation between represented objects and real-world objects, semiotics has addressed the issue of realism on the one hand in terms of “referent’s illusion” (Greimas and Courtés 1979, p. 178) and on the other one through the Peircean notions of “icon” and “index”. However, it is impossible to recapture here, even briefly, what is known within semiotics as the “iconism debate” or the discussion around the linguistic theory of enunciation used to address the issue of referentiality. What is relevant to this article is that this debate pointed out that any perceived similarity is an impression that arises from satisfied *expectations* about aspects deemed *pertinent* (Eco 1968, p.114; Prieto 1975).

Consequently, and to conclude, realism is an *effect of reality* that stems from the semiotic structures (mixing the cultural and the cognitive) that we use to grasp and make sense of reality in terms of attributing meaning and identity through contextually applied *interpretative criteria of realness derived from our habits*.

Returning to our main object of study, there is no a-priori reason to exclude the face as an object semiotically influenced by cognition and, in turn, this implies that face recognition is actually a deeply inferential (deductive) and interpretive process. Indeed, while a realistic face is one that is “exact, natural, and expressive” (Zikky et al. 2013) from a semiotic perspective each of these key features is not merely a matter of perception but rather a matter of interpretation that occurs in both physical and virtual contexts. Simply put, phenomenologically recognizing a face as real implies the application of different criteria of realness that will then be applied across the board to artificial faces judged to be more or less realistic. Moreover, such interpretation is not only a matter of observation but also a pragmatic matter of interactions: these criteria depend strongly on what we do with faces and what such faces can do to us. From this perspective a real face is any *face-looking* thing which is able to reproduce the full complexity of the *experience* that we have every day by interacting with the faces of others as well as by using our own face in a variety of situations and for a multiplicity of purposes.

If the emotional agency of this face is capable of moving us with its expressed sorrow, if it is able to trigger love at first sight or *first impressions* (Todorov 2017), if behind its expressions we can intuitively read and misread feelings and thoughts, and if its features can make us naturally recognize traces of both subjectivity (the human’s uniqueness) and intersubjectivity (the human’s socio-cultural belonging, a certain *familiarity*), if its physical features (wrinkles, moles, sunspots, cracked lips) are detailed enough to infer through them a subject’s life story, then inside such paradigm this face is not simply realistic but can be defined as real because our *relation* with it will not be experienced as artificial (Leone 2021a, b, c, p. 12). Thus, within our semiotic paradigm, the realism of a digital face can be studied in terms of a face-looking *surface of expression* which is able to trigger an *effect of reality* by enacting all the perceptive and cognitive *habits* that its interpreter has developed toward physical faces during his life and which determined the very criteria of realness used when recognizing something as realistic. Realism becomes a matter of potentials positively enacted in a given context and by a culturally situated human being, not of essential properties of a given isolated object. If a digitally created face is *potentially* able to enact anything that we *would* do/think/feel in front a physical one, then this face becomes semiotically real (Peirce 1992, EP 2.456). Hence, the possibility of studying the realism of a digital face as an effect of reality depending on the relation between the object’s cognitive affordances and the subject’s habits. Such habits will be based here on five main pragmatic criteria:

1. Being a *face-looking* thing from a neurological and unconscious point of view: the capacity of such object to activate face-selective neurons and face-specific brain regions in the *detection* of faces.
2. Being a *face-looking* thing from a cognitive conscious standpoint: *recognizing* something as a face, associating it with an identity and willingly engaging in specific interactions (e.g., gaze avoidance).
3. Being a *face-meaning* surface of expression from a cultural conscious perspective: attributing meaning to such face on the basis of an explicit culturally shared *knowledge* (e.g., she is Asian)
4. Being a *face-meaning* surface of expression from a cultural unconscious point of view: the capacity of such object to activate culturally *biased* assertions without the subject being fully aware of them.
5. Being a *face-acting* surface of expression which, based on the previous four points, has different forms of agency on a subject: from arousal to facial expression mirroring (Dimberg et al. 2000a, b).

Each of these involves the activation of cognitive and semiotic processes that rely on prior knowledge of faces. Such prior knowledge, Peirce’s *habits,* is developed both genetically, historically, and during the subject’s lifetime through interactions with faces. With 1 and 4 being in Peircean terms *beliefs*, a form of pre-cognitive understanding based on guessing habits (Vecchio 2020) that can only be verified and questioned in retrospect. And with 2 and 3 being a *knowledge* which is instead built on a form of explicit reasoning (Idem) which can be described with relative ease (e.g.,: this is a face because there are two eyes, a mouth and a nose).

When all the criteria are met the object in question is both fully believed and fully recognized, enacting in this way all the real-life pragmatics (by both looking as a face and *acting* on us as such) of face meaning-making and consequently causing us to establish a relationship with the object that is perceived as real regardless of its materiality and ontogeny. Otherwise, if some of these criteria are not satisfied, we will have different degrees of realism. In relation to the previous criteria, the realism of a CG face can thus be studied in terms of:

1. Detection and misdetection (e.g. pareidolias)
2. Identification and misidentification (e.g. mistaken identities)
3. Cultural reading and misreading (from biased habits to facial deception)
4. Impact of all the previous elements on the viewer’s cognition and emotions

This approach, belonging to the pragmatic legacy of semiotics (Paolucci 2018), will be the one we chose for four main reasons. First of all, because metahuman faces are cultural artifacts created for communicative purposes aiming to reach a status in which they can lie to us. A type of object of study which is perfect for the semiotic metalanguage and methodology that, historically, have been developed precisely to analyze “anything which can be used to lie” (Eco 1975). Second, because facial recognition and interpretation is deeply rooted in cognitive mechanisms, and semiotics is extremely complementary and congenial with cognitive studies (Paolucci 2021). Indeed, in the second part of this paper we will refer to several well-known cognitive phenomena of face perception going exactly in the direction of the beforehand exposed semiotic perspective. Third, because the newest development in visual semiotics (Dondero 2020) has postulated a fundamental distinction between the content (*apport*) and container (*support*) of images, highlighting how meaning-making is the result of the viewer’s relation with both. This difference is of fundamental importance in the study of objects such as virtual faces, which present the issue of understanding differences and similarities on these two separate levels. Fourth and finally, because in the last two years semiotics has already been applied to artificial faces in numerous studies (Leone 2021a) and has thoroughly proved the efficacy of a critical stance toward the concept of a “natural face”.

So, hoping to have clearly defined our epistemological framework, we can now start our analysis.

## Technological Causes of Realism: AI Managed 3D Scanning and Motion Capture

To understand the experienced realism of MHC, the first aspect to examine is how these faces are made. There are actually very obvious technical reasons behind any contemporary achievement of digital realism. Some of these are related to physical context (place, light, ambient noise), some are related to the hardware (screens and their parameters) and finally, some are related to the techniques developed for and integrated into MHC. Taking into account all these factors would, however, require much more space than we have here. It would also require a partially different theoretical background related to the specific semiotics of media experience (Eugeni 2010) and devices producing effects of virtual realism (Eugeni and Catricalà 2020). Coherently with our research question and theoretical framework, we will limit ourselves to briefly highlight the two main technologies at work in MHC that could have a critical impact on realism: 3D scanning and AI-Driven motion capture. These were selected for two distinct reasons. First of all, they are the one recognized by the developers as the game changing features of MHC^8^ and of its engine.^9^ Second, these technologies have the same purpose of grasping real-world data to produce realistic digital faces through automatized processes. Indeed, the believability of MetaHumans comes first of all from the scanned nature of the data which is “sourced from actual, real, plausible human faces”^10^ through “custom-built scanners”^11^ to explicitly create an effect of believability^12^ in terms of physical plausibility.^13^

Without going too much into details, the main value of techniques such as 3D scanning is the capacity to measure the different profiles of an object in terms of deformation points and consequently to allow for highly variable objects (such as faces) to be grasped with great precision. As such, the realism of these faces depends on the highly photorealistic nature of its database containing high-definition source material that has on the one hand a high complexity and geometric precision, and on the other hand a considerable amount of visual data corresponding to the multiple aspects of our perceptual grasp of a phenomenological reality. This qualitative and quantitative feature is directly related to the possibility of mass production of photorealistic digital humans that must be as diverse as possible to simulate their uniqueness, which is why increasing the database is deemed a priority by the creators.^14^

The scanned data is however not sufficient to achieve the experience of dynamic facial expressivity, which is why MHC includes and is based on the latest technology of motion capture. Similarly to scanning, *mocap* is also a technique aiming to automatize the grasping and reproduction of facial data although it is mostly focused on facial movement. A technique which is far from new but has constantly been constantly improved over the last years (Zikky et al. 2013).

However, both technologies would be incomplete without the work of artificial intelligence, and especially of deep learning,^15^ that adds a third layer of non-human mediation in the process of producing realistic digital faces. It is a combination that did not come about specifically with the MetaHuman project but that was part of the broader R&D on digital realism. MetaHumans are consequently also *deephumans*: the result of the last thirty years of research on computational facial recognition methods and artificial intelligence with the turning point of deep learning (Arcagni 2018; Le Cun 2019). In this way, Epic follows the tendency^16^ of digitizing and transducing the real through AI rather than representing it from scratch. Thus, while the unprecedented realism of faces in a movie like *Final Fantasy: Spirit Within* (2001) came from a raw capture of reality which was then heavily refined and mediated by humans,^17^ the new realism of the metahuman faces seems to work in the opposite way and seems to introduce in the world of computer-generated faces the same differences that existed between painting and photography (Leone 2021a, b, c, p. 10).

## Comparative Semio-Anatomy: Human VS CG Faces

Now we know the main technologies behind the MHC faces, but what do these scanned, AI-generated faces actually look like? Indeed, even though we have seen that the main value of MHC lies precisely in avoiding any process of creating faces from scratch, the resulting object will still be analogous to digital faces created by other means.

Since from a semiotic perspective the effect of reality depends on interpretative possibilities offered by a certain object, to answer our two main research queries we need how digital faces differ from physical ones. We will now briefly compare them^18^ and present to our readers (via the links in the footnotes) some visual examples of the structure behind MHC faces.

While a physical human face is made of craniofacial muscles lying underneath the skin and originating from a genetically determined skull, the main shape of a digital face is determined through modelling/sculpting a polygonal *mesh*^19^ (a geometrical structure made of vertices, edges and faces^20^) and its static appearance depends on a *texture* (the face’s “image”) applied to it.^21^ To achieve a strong effect of realism, a “UV map” is created to apply the 2D texture to the 3D model^22^ by creating matches between pixels and vertices.

In addition, modelling such mesh also entails the creation of a *topology* mimicking the human muscles and creating a facial system based on *differences*. Here distinct parts of the face constitute different sets of circularly connected edges called “loops” which are then interconnected.^23^ The realism of the texture itself is given by highly detailed images and by the presence of several *layers* (or *levels of details,* from now on *LoD*) that mimics the phenomenological and anatomical complexity of real faces and especially of the outer skin.^24^ Finally, three key parts of the face are also added: eyes, teeth and hairs.^25^ Eyes exist in MHC as texture presets composed of iris and sclera with highly configurable properties. Differently, teeth are added as 3D objects composed of “control points” that can be adjusted and each corresponds to distinct parts of the teeth with a strong emphasis on the possibility of variations. Lastly, hair is added as “groom assets” that “can be made up of different types of geometry from individual strands (allowing for cinematographic quality) to cards to a low-poly mesh” and thus allowing different *LoD*. In some cases, such as the eyebrows, hairs can be simply added as an extra layer of texture, in others they are a standalone object allowing the pinning of every hair to a precise position on the head. This is especially important since the realism of the hair (or groom) depends mainly on detail and variation, and therefore a fundamental work is needed to implement aspects of asymmetry and imperfections (called “noise”) in the distributions of such hair to create an effect of realism by breaking uniformity.^26^

With all this we have now created a very realistic 3D face, but such object would still be far from what metahuman faces actually are. In fact, so far we have created a static and non-animated face, whereas metahuman faces have a strong focus on facial animation and expression. From this standpoint, one of the key semiotic features of MHC is the fact that such faces are designed and programmed to be coupled with a digital environment mimicking physics. Metahuman faces look real because they are everchanging objects which can be deformed on several aspects: from hair movement to lighting^27^ and aging.^28^ Such facial deformation is not limited to the environment but also affects expressiveness. So, how do metahuman faces dynamically express emotions and recreate motion stimuli? In the previous paragraph we have already mentioned the key role of motion capture in doing this, but we can look even deeper at how a face model can be digitally animated and thus express emotions. In the case of digital faces, anything expressed has obviously nothing to do with muscular contraction and facial nerves but is the result of a process named *rigging* in which functional *joints (*connections between parts*)* or *blend shapes* (combination of facial deformations) are created to animate the mesh.

As a result, expressive equivalences can be created between muscle movements, such as the *procerus* muscle used to express anger, and mesh deformations/animations which will directly replicate the effects of the muscle, such as moving the eyebrows downward to express anger. Contemporary software of CG face creation, such as *Character Creator 3*, offers standard templates (premade shapes/deformations) of facial expressions^29^ that can be further adjusted and customized^30^ by acting on each single part of the face’s model. This succession of deformations is therefore how a face can be recognized as expressive and can be done either manually by an artist or by AI-powered scanning and capture technologies. Finally, in the specific case of MHC all these different expressions depend on different parts of the rig (interconnected bones) that interact together (moving the jaw will deform all the rest of the face) and can be automatized through *triggers* and *tags*^31^ to occur in a given situation.

We can therefore see that, in ontological terms, metahuman faces could not be more different from our own. Whereas the organicity of the human face determines both its predetermined mutability (with skin, muscles and even bones aging and changing the shape) and non-mutability (depending on the face that we are born with, some different faces will not be possible to have), the mathematical nature of the metahuman face allows for both predetermined immutability (it is eternal) and all-mutability (any face can become any other since a change requires only a mathematical manipulation). Additionally, whereas real human faces exhibit some patterns of movement that are more or less cross—culturally (Ekman 1970, 1994; Russell 1995) attributed to emotions, in contrast the movements of CG faces are triggered through predefined behaviors depending only on the creator’s desires: something like “wide eyes, raised eyebrows, open mouth, chin pulled in, head down” could be programmed not only to occur each time that the character is bored, but also to occur only in one specific character. Finally, the texture of digital faces is fundamentally a single, all-encompassing, object that is the face itself and has the *skin* as the deeper level onto which various independent elements can be added (wrinkles, moles, scars, capillaries). Differently, a physical human face is largely a reflection and result of what lies deep within it. This is at the opposite of what occurs when modifying a metahuman face in which it is possible to act directly on the *level of expression* to produce the desired communicative effect. Digital faces are therefore made of interconnected parts which are however not necessarily a whole. This results in a form of *hollowness* which is rather literal in the cases of occasional glitches and bugs^32^ making the faces of digital avatar only partially disappear.

However, these differences are mostly invisible to the human viewers who has only access to the outer/superior *level of expression* of the face. A face that, as we have seen, is actually a surface of differences and relations creating meaning though its *content* and transformative capacities. Such face is also highly likely to mimic all the behaviors and physical features of human visages and facial expressions, consequently allowing for a strong effect of reality and with most of the unrealistic features (such as an excessive symmetry), depending on the human creator.

Knowing the main characteristics of both the technology behind MHC and the concrete face-objects produced by such software, it is now time to switch side and look at what is likely to occur in the mind of a human when faced with a metahuman face.

## Metahuman Faces Between Cognitive Studies and Semiotics

As accurate as MHC features may seem, we have seen that any recognized “realism” is always the result of a semiotic interpretation born from a complex visual and cognitive experience. The question, then, is whether metahuman faces are likely to meet the previously criteria of realness exposed in section two, distinguishing themselves from previous CG faces in terms of realism. Although only empirical researches can prove whether MetaHuman actually triggers most cognitive and perceptual processing, it is nonetheless possible to hypothesize the answer through a semiotic reflection. To do so, let us first briefly resume the main characteristics that were observed in the previous sections:

A. The non-direct human mediation in the creation process of such visages through representations allowing for unprecedented mathematical and geometrical accuracy.
B. The extensive database of the scanned data allowing for almost countless combinations.
C. The high level of details of the photorealistic represented facial features.
D. The replication of faces as dynamic and transformable surfaces.

With this in mind, in this section we will now examine the potential impact of A, B, C and D on the previously explained interpretative criteria of realness involved in grounding face-specific effects of reality.

### Consequences of Mathematical Accuracy: Hyper-iconism

Consistent with the theory of semiotics set forth above, to reconstruct the realism (*effect of reality*) of metahuman faces we have to understand how do they satisfy the *beliefs* of their viewers. In this regard, geometric exactitude is somehow the first and most obvious expectation and aspect of any face-realism, in some way its literal meaning (Eco 1979), so much that an ideology of the face has been funded on its measurability (Leone 2021a, b, c). The answers to why such primacy of the face shape exists can be found in the cognitive studies on face perception, which through the case of face pareidolia (Palmer and Clifford 2020) have demonstrated how a t-shaped spatial configuration (Tsao and Livingstone 2008) can activate a knowledge about faces that is genetically inscribed, disregarding whether this face is real, a picture, and even if it is a face. Consequently, if such activation occurs even in not particularly accurate face-objects, we have little reason to doubt that metahuman faces can fulfill this condition. Now, even from a purely neurocognitive standpoint, faces are actually much more than t-shaped configurations. As an example, it has been very recently demonstrated that robot faces, despite having such shape, are dissimilar enough from human faces to be processed differently (Geiger and Balas 2021) and thus to be also consciously perceived as partially unreal. On this point, however, the same study also showed that human-like inanimate faces (CG faces and dolls) are processed much like real faces.

What is more interesting for us then is to reflect on the specific causes of such accuracy, namely the scanning and automation process involved in the production of metahuman faces. Indeed, here a second notion from Peirce’s semiotics comes into play: that of *indexes*. Indexes are signs that are endorsed with high trustfulness not because of their similarity with their objects, but because they are produced through their *factual connection* with them, often in terms of spatial and temporal contiguity, and therefore are logically interpreted as *traces*: with the prototypical example being the footprint in the sand. Clearly such traces are once more not intrinsically true (Leone 2021a, b, c) since the causal relationship implied depends on prior knowledge which can lead us to wrong interpretations (the footprint could have been drawn on the sand). But what we are interested in here is how, historically, higher impressions of realism in representational object are strongly connected with the development of automatized technologies capable of grasping an item in indexical terms: with the most notorious example being the one of photographs which are iconic indexes and present an indexicality deriving “from their physical connection (via light) with the represented objects” (Sadowski 2016). From this standpoint, the newest AI-enhanced scanning technology constitute the highest degrees of indexicality possible with each metahuman face being a visual conglomerate of indexes. Something that will most likely have an impact on the neuro-socio-cognitive processes of mirroring/mimicking, which in the case of emotions rely precisely on the possibility to read a wide range of facial configurations without substantial distinction between reality and representation (Leslie et al. 2004). By having such a high-degree of indexicality, metahuman faces may therefore enact not only a sense of *belief* from the point of view of face-detection but also a sense of *trust* also from the point of view of the impact of other’s faces in our cognitive activity and consequently on the viewer’s emotions and “gut reactions” (Prinz 2006).

In conclusion, 3D scanning, mocap and AI processing are at the root of the experienced realism since they work indexically on real faces to create enhanced icons (“hyper-icons” in Volli 2020) through transduction (Paolucci 2010) of phenomenological aspects of them. Therefore, allowing us to have access to the meaning of faces through the “mathematical truth” behind our perceptive experience of every day’s faces.

### Consequences of the Database: Sociocultural Believability

Being consciously aware of being in front of a face is what allows us to understand the phenomenon of pareidolia and to communicate with others. To do this we need to recognize on an object the *features* belonging to the knowledge about what a face is. Here, a face-looking thing will need to include some iconic and minimal semantic elements such as the eyes, nose, and mouth. From a cognitive point of view, such object recognition (DiCarlo et al. 2012) is related to the early stage of visual processing (such as edge detection) influenced by higher level representations related to prior knowledge (Teufel et al. 2018).

Even more interestingly, exactly as with the case of pareidolia and accordingly with Peirce’s theory, our prior knowledge (*habit*) of the face seems to undermine our direct perception of the object itself also at this level. This is demonstrated by experiments regarding both the lesser accuracy of inverted face recognition (Sekuler et al. 2004) and the hypercorrection of inverted eyes in the Thatcher’s illusion (Thompson 1980). Therefore, once again here the photorealism and accuracy of MHC is well above what is needed to cognitively and consciously ascribe the status of “face” to a thing: let us think at the unrealistic visages of abstract art^33^ which sometime are not even presenting a proper “T-shape”. From a semiotic point of view, we thus have a knowledge and habit for which in front of a face we somehow perceive and believe our own construction of the object. This knowledge is a repertory belonging as much to our direct experience of the world as to the general social encyclopedia of texts, such movies but also novels (Magli 2016), that have exposed us to faces. Through such exposures, we have the creation of Peirce’s *types* through principles of generality (Niklas 2020): classes of *abstract* objects that belongs to the notion of face. In the case of eyes, we can for example think of differences such as “round eyes” vs “almond eyes”, categories which both regroup and distinguish millions of people. It is likely that these types play a key role in the cognitive process of identifying and remembering faces by relying on our memory (Lopatina et al. 2018). Indeed, for something to be not just *a* face but *someone’s* face, we need to be able to grasp specific facial features to classify and categorize them. These sets of features are how we distinguish not only “Jhon” from “Paul” but also how we apply to a person, through stereotypes, any semantic categories regarding the gender, age, race and so on. These configurations of *types* play a key role in the agency of the face both in terms of what we may consider familiar (Meike and Ida 2018), with such familiarity being observable even at a neurocognitive level (Zhou et al. 2018), and in terms of face impressions (Todorov 2017) related to faces that we may or may not intuitively trust. In MHC we have seen that the variety of the scanned database in terms of presets allows for a diversity of facial types just in this way. The database meets the expectations of our cognitive repertoire. Moreover, the variety of facial *types* also allows us to read faces as objects which inevitably display traces of the subject’s constitutive intersubjectivity and sociality: this occurs each time that we find similarities between father and son, among people belonging to the same country, and even sharing similar socio-cultural backgrounds (think of the stereotypes of professors, soldiers, etc.).

Indeed, what makes a face real is our intuitive propensity to produce through them various acts of culturalized semantic attribution such as “this is Sun, she is an Asian, happy, young, sincere, and beautiful”. Attributions which are however still fundamentally a fallible interpretation responding to the general processes through which humans make sense of the world. To take a simple example, inferring Sun’s happiness and serenity from her smile is a *guess*: it could actually be a lie, a way to mask her depression or express the fact that she about to murder us. Not only that, but our own perception of Sun’s happiness could easily be biased for cultural reasons as recent studies on emotion perception have proved (Korb and Massaccesi 2020; Korb et al. 2021). Indeed, the fact that we have a natural tendency to read faces does not correspond at all to the fact that we are good as such activity, it is actually quite the opposite (Evans et al. 2016). Interacting with a face is a conscious bet between what we intuitively believe and what we know. The accuracy of facial interpretation is in this sense no different from the way in which we interpret other objects: the correlation between something expressed and its meaning is highly dependent on the interpreter’s habits and the historical construction of what something like a smile can mean (Rozenblatt, 2016). On that note, MHC not only allows for such socio-cultural recognition to occur but is entirely based on such possibility by combining scanned “parts” of different individuals and even including a function to create a particular face from its relationship with others.^34^

The database therefore allows MHC to meet not only the second criterion of realness through identification (and misidentification) but also the cultural (three and four) conditions of face believability in terms of sociocultural meaningfulness by letting us identify faces that we can endorse with intersubjectivity. Once again, however, MHC possibilities are far above what is actually need for such cognitive and semiotic process to be triggered. In fact, the possibility and relevance of correlating visages-types with meaning is nothing new in the field of computer graphics and even more specially in the history of videogames characters (Giuliana 2021). Using face-types to attribute a culturally connotated identity and to infer social relationship between subjects was for example already possible in the 1983 game “Mario Bros” by comparing Luigi and Mario.^35^ It is consequently only when combining this database with the quality (*LoD)* and quantity of each single item-type (next paragraph), that we can understand the true novelty of MHC.

### Consequences of LoD: From Familiarity to Ambiguity

In Peirce's theory of meaning, “types” are distinguished from “tokens”. The distinction between a type and its tokens “is an ontological one between a general sort of thing and its particular concrete instances. […] Types are generally said to be abstract and unique; tokens are concrete particulars […]” (Wetzel 2018). In a sense, types are essential to reduce the amount of effort required to grasp the phenomenological complexity of a world presenting countless tokens. Consequently, a realistic face cannot limit itself to have *types* of facial features but must present *tokens*: someone’s face has not simply an “adult straight Greek” nose but has what is perceived as that person’s specific nose. Now, perceiving the unicity of a given facial feature depends on our perceptual capacity to grasp reality as something extremely detailed in terms of color gradients, millimetric differences in length and width, etc. Here, the mathematical nature and accuracy of metahuman faces can only allow such a level of details that, in turns, corresponds to a *high-density of information* that is captured and interpreted by the viewers. The first obvious consequence of such information is an effect of realism in terms of the digital face *materiality* looking extremely similar to physical faces because of similar fine perceptual processes triggered, despite its completely different nature. The second, perhaps less obvious difference, regards the agency of such information on us. Indeed, the fundamental role of faces as containers of information is well-known and has been even more highlighted by the recent issue of facial masks due to Covid-19 which results in loss of trustworthiness (Marini et al. 2021). In this sense, we can infer that dealing with faces which have an insufficient amount of information is quite an unnatural and uncomfortable situation for the human viewer. We may also hypothesize that many uncanny effects of artificial faces (Masahiro 2012) stem from face-objects which are detailed enough to trigger a non-merely type-form of face-knowledge but are however not accurate enough to be read as true tokens. As an example, a low resolution black and white picture is much less likely to disturb us in terms of looking unreal (as in the cases of composite portraits) but is also less likely to convince us of its accuracy in comparison to the work of a GAN. Similarly, some heavily distorted faces (as in the case of Japan’s *anime*) do not disturb us as much as some CG photorealistic faces (Schwind et al. 2018). From this perspective, if we compare the previously referenced pictures of MHC with the best-looking photo-realistic CGI faces of the 1990s, the gap between the amount of information is quite clear.^36^ Being highly informative, metahuman faces are consequently highly readable and can reach a degree of communicative complexity for which misreading is also likely to occur. Furthermore, the mathematical accuracy combined with such LoD can only increase the capacity of such faces to enact effects of familiarity. Finally, such quantitative increase in information may also replicate realistic effects of intuitive ambiguity in the attribution of meaning and reading of emotions.

### Consequences of Transformation: Holistic Multidimensionality

The last remaining characteristic of MHC is related to the capacity for such faces to change under different situations, in other words to have a *temporality* both on a synchronic level (such as facial expressions) and diachronic one (such as the traces of ageing).

In absolute terms, this is obviously not something inherently new: any kind of animated artificial face present a certain degree of deformation to obtain a desired effect of expressiveness. From this point of view, Walt Disney’s 1937 “Snow White” exhibits the same temporal capacity of MHC. The difference comes from the fact that in MHC such a transformation potentially effects every single pixel of the face (due to its mathematical nature) through a wide range of variations (due to LoD and database) rendered in real time through the computational processing power of today. In this way, an unprecedented number of different *discrete* and non-generally typological (e.g.: angry face, sad face, etc.) deformations are made possible, allowing for complex distinction in facial expression (such as the Duchenne’s smile) and with each one of them changing the *overall* relationship of the elements appearing on the level of expression of the artificial face.

This “shapeshifting” capacity of metahuman faces plays a critical role in the attribution of realism for two distinct reasons. Firstly, it makes a connection between the spectator’s knowledge on faces and the one about the physical world. Indeed, a believable face must be consistent with our knowledge and *habit* about how, for example, light works on real surfaces. In doing so, we semiotically recognize metahuman faces as belonging to our same *possible world* (Eco 1979). This is why light reflection is such a great deal in contemporary virtual realities (think of ray tracing) and also why the technique of “skin shading”^37^ is a key feature in creating impressions of reality when creating a digital face. Now, because metahuman faces are highly informative objects designed to be used during various types of interactions and physical contexts, they allow a remarkably high degree of realism by allowing us to enact many of our habits concerning the face as a dynamic and changing object of the world. Secondly, it creates the requirement of an *effort* and uncertainty about the face. This second feature is undoubtedly realistic since experiencing a real face in the physical world is not simply recognizing a great quantity of perceptive information about it, but it is also witnessing such information changing under different conditions: which constitute the multidimensionality of the face as a complex item of the world and involves difficulties well known in the field of machine facial recognition (Lee-Morrison 2019).

This is especially true in reference to the fact that human face recognition also entails the process of *not* grasping the face as a set of distinct and specific parts but instead of *guessing* it in holistic terms. In fact, it is this holistic aspect that has influenced some of the modern models of face recognition by shifting the focus from a sum of parts to a “whole” because “individual features and their immediate relationships comprise an insufficient representation to account for the performance of adult human face identification.” (Lee-Morrison 2019, p.61). In the cognitive sciences, this holistic aspect of face recognition is named “configural processing” and encompasses various aspects (Maurer et al. 2002) that are of great value as they seem to convincingly explain^38^ many phenomena of distorted face perception such as in the case of face inversion (Van Belle et al. 2010) and the composite face illusion (Murphy et al. 2017). Returning to semiotics, this implies that even the cognitive and interpretative process of face recognition itself seems to follow a classic interpretative path: “Eigenface is based on the premise that the most relevant information about an individual face has to do with the ways it is different from another.” (Lee-Morrison 2019, p. 66). On this point, MHC may raise these possibilities of holistic variations both through the quantity of the database and the LoD of each elements.

## Contextual and Intentional Limits of MHC’s Expressivity

In light of this overview, the potential of MHC should allow unprecedented effects of realism in terms of the facial expressivity of metahuman faces and of their agency on viewers. Yet, this also represents perhaps the hardest test to pass since even physical faces may be judged as more or less “convincing” when they replicate an emotion (as YouTube demonstrates with, for example, lists^39^ of best and worst crying scenes in movies). The quantitative, qualitative and transformative qualities of MHC artificial faces are therefore necessary but not sufficient on their own to ensure the actual credibility of such expressions. This last aspect introduces a must needed doubt about the actual experiential realism of metahuman faces. In fact, we have seen that even the creators themselves admit that such faces do not succeed in trespassing the uncanny valley. Which is not so difficult to conceive if we take in consideration not only the diverse semiotic *container* (a screen) and *situations* of interactions with metahuman faces, but if we also consider the different *intentionality* toward CG faces. Indeed, even in digital games it is quite rare that the misreading of CG-generated faces has a negative impact on the player (Giuliana 2021). Thus, even though metahumans are complex enough objects to allow for an intuitive distinction between a fake smile and a genuine one, this complexity is wasted without a context that offers some reason to question whether a smile is potentially fake. In fact, we all know from personal experience how much prior intentions and beliefs toward a person have a high probability of affecting both the interpretation of its facial expressions and our attention to them. This brings us to the problem of perceived realism itself. Indeed, from a cultural standpoint, realism is an effect perceived and believed by the interpreter. And interpretations are never really separated from the socio-cultural context in which they occur (Leone 2021a, b, c, p. 10). A context that in semiotic terms can be conceived as a network of all cultural texts (Lotman 2006) containing CG faces, and that in philosophical terms can be conceived as an epistemic environment (Blake-Turner 2020) in relation to the impact and commonality of artificial faces in our lives. Realism is, in fact, a complex impression which goes far beyond the mere capacity of a medium to “trick” perception. For example, whereas many know the famous anecdote about the audience running away from Lumière’s train, few know that early color films were judged less realistic than the black and white ones (Stam 2000). From this point of view, the realism experienced in front of digital faces can hardly be completely separated from the acquaintance with not only digital technology but even more specifically with 3D CG-faces. Therefore, it would be a mistake to examine the social discourses about the realism of MHC without considering how much often we are exposed to CG artifacts today. In fact, more than a decade ago several scholars could already speak of “digital” (Creeber & Martin 2009) or “software” (Manovich 2010) culture, and it is now almost thirty years since we are exposed to digital faces through the products of mass-culture. Let us think of the first full CGI films and series for children (Toy Story 1995; Reboot 1994) and of course at digital games of world-wide success with 3D characters (Super Mario 64 1996; Final Fantasy VII 1997). Up to the production of *Final Fantasy: Sprits Within* (2001), which was “the first entirely computer animated, photorealistic feature-length film based on the principles of live action cinema” (Monnet 2004, p. 97) and which, despite featuring faces significantly inferior to the one of MHC, created similar reactions: “In the past few weeks, how many people have you heard ask the question, “What do you mean those actors aren't real?” when referring to the *Final Fantasy* trailer? During the movie did you occasionally forget that it was all CGI?”.^40^ This issue of the cultural acquaintance with digital faces can be understood inside our reflection from a cognitive standpoint in terms of familiarity but is for the most part independent from the software itself. So, none of what we have exposed in the previous section should be enough to postulate a somehow causal connection between the highlighted causes of the higher effect of reality and the actual capacity of MHC to create faces which are believed as real and able to lie.

## Conclusions

To conclude, it is now time to answer to our two research queries. Our analysis has shown that, despite some minor exceptions, metahuman faces should be able to trigger/enact most of the major habits related to face detection, identification, reading and agency in a significantly different way than previous CG faces. We can therefore affirm that MHC faces actually constitute, from an academic standpoint, a different type of artificial faces characterized by a qualitatively superior effect of reality.

Summing-up our research, we have found that the causes behind such realism are the following ones:

1. The AI enhanced motion cap and 3D scanning techniques used in MHC are methods of production that allow such artificial faces to possess a geometrical accuracy far above the necessary requirement to trigger face detection habits and to enact, through indexicality, a physical plausibility on the level of the subject’s *beliefs* and which should be able to endorse metahuman faces with agency from the perspective of the neurocognitive impact of facial configurations and expressions.
2. The scanned nature and the (potentially ever-expanding) variety of the database comprise a repertory of facial elements working as *types* which are at the heart of processes of identification and culturalized readings in terms of singular recognition (uniqueness of a face) and intersubjective recognition working on the level of *knowledge*. Moreover, the intersubjective dimension of metahuman faces should also have an agency on the viewer by triggering unconscious cognitive processes of *familiarity*.
3. The high level of details of the database elements can be understood as a mean by which metahuman faces are endowed with *high-density of information*, granting to the viewer’s the possibility to phenomenologically process the digital *materiality* similarly to the one of physical faces and to recognize not merely facial *types* but also facial *tokens*. This, in turn, enhances on the one hand the effects of identification seen in the previous point, but on the other hand also opens up the possibility of semantic ambiguity and communicative complexity by making the reading of such faces less obvious and more realistically fallible.
4. The transformative capabilities of MHC grant such faces the fundamental aspect of *temporality* and *possibility*, further increasing the complexity of face recognition and reading. But more importantly, they reflect the impossibility of reducing faces to a simple one-dimensional item, therefore enacting habits of face meaning-making under the form of an *effort*.

Finally, we found that the real novelty of MHC lies in the way each of these four points is interconnected and influence each other. And that, despite their actual potential to endorse a face with a superior effect of reality, the actually experienced realism will still depend on external factors related to intentionality and cultural context.

Coherently with our results, we will now highlight four future lines of interdisciplinary research:

A. The first line of research concerns the relation between the techniques of digital face generation and their perception. Indeed, we have seen that the increased realism comes first of all from a form of mathematical accuracy deriving from techniques of facial captures and representation in which human mediation is less and less involved. It would be interesting to study the impact of different techniques of face creation on the cognitive and psychological processes involved in both the attribution of realism and of meaning. Especially in terms of studying the differences between techniques based on mostly human-mediated representation and mostly AI mediated representation. Furthermore, since we have shown on many occasions that face perception is far from being a mere question of neutral perception, it would be interesting to test the thresholds of such mathematical accuracy. For example, would we really perceive any gap of realism in an infinitesimally less accurate mathematical model of a face? Is there a boundary or threshold of realism in mathematical and geometrical terms?
B. The second line of research is about digital faces as containers of information. First of all, is there really a quantitative causality between the amount of information and the impression of truthfulness? As an example, we have seen that hairs can be implemented on different levels of details ranging from being textures (lower amount of information) to being 3D objects (higher amount of information). These differences could be tested. Also, another inquiry could be made on the possible differences between facial parts in terms of weight on the attribution of meaning to understand when and under which conditions does the token-effect appears.
C. The third line of research moves from the face as an isolated object of perception to the face as a situated object of interaction. Here the first question is whether or not face recognition and meaning attribution processes, from early perception to high cognition, are influenced by contextual and pragmatic elements. For example, do a same face can be interpreted differently when put in an interactive narration such as in the case of a videogame rather than in a movie trailer or in a customer service such as Uneeq^41^? Do we perceive equally a same face when it is isolated and when it is a crowd of other digital faces? Do we attribute more realism to a lesser accurate face model in a more accurate context of lighting or is it vice-versa? Do technologies such as Virtual Reality, which changes our overall sensorial context of perception, have an influence on the attribution of realism? The second issue, differently, regards the social and historical context. Here the main query is about the relation between the acquaintance with digital faces and their perception: do we tend to perceive digital faces as more realistic if we are used to interact with them and or are exposed to them for extended periods of time? Does the epistemological crisis of the face due to objects such has deepfakes influences also the representations of non-digital faces?
D. The fourth and last line of research regards the already existing researches on the differences between real and artificial faces from the point of view of human perception. On this topic, scholars such as Ben Balas have extensively and continuously worked (Balas 2012, 2013, 2014, 2015, 2017) but without using software such as MHC and by generally referring to “artificial” faces as a class of objects. Our paper indicates that it might be interesting to replicate past experiments made with software such as FaceGen 3D Modeler on faces created via MHC to see if any differences can emerge.

These lines of research are all semiotically oriented since they focus on the issue of meaning-making in terms of construed differences, yet they require methodologies and epistemologies that do not belong to semiotics itself (Viola 2021). Finally, given the transdisciplinary vocation of this discipline, we sincerely believe that such semiotically oriented lines of research can shed a light on critical issues that do not belong to semiotics itself. Chief among them is that they certainly raise doubts about the interpretative equivalence between faces in flesh and artificial ones. A doubt that undeniably deserve an answer given the increasingly common usage of CG faces in all sort of scientific researches that could be compromised precisely by neglecting the peculiar interpretative aspects of CG-face identification, recognition and reading.

## Footnotes

1 https://www.youtube.com/watch?v=1tjkSpoa7V8.

2 https://www.unrealengine.com/en-US/digital-humans.

3 https://www.unrealengine.com/en-US/blog/introducing-the-matrix-awakens-an-unreal-engine-5-experience.

4 https://www.youtube.com/watch?v=qvnJ_wBiKUI.

5 https://www.gamespot.com/articles/epics-new-MetaHuman-browser-app-lets-you-make-photorealistic-humans/1100-6487339/.

6 https://www.gamesindustry.biz/articles/2021-02-10-epics-new-tool-promises-high-fidelity-human-characters-in-under-an-hour.

7 Experiments of both optical (from Muller-Lyon’s lines to the Ames room), auditory (from Franssen effect to McGurk Effect) and even tactile (rubber-hand experiment) illusions.

8 https://www.youtube.com/watch?v=HuAAdsZPLlE.

9 https://www.youtube.com/watch?v=qvnJ_wBiKUI.

10 https://www.gamesindustry.biz/articles/2021-02-10-epics-new-tool-promises-high-fidelity-human-characters-in-under-an-hour.

11 https://www.gamesradar.com/has-epic-transformed-character-creation-for-good-with-MetaHumans/.

12 Idem.

13 https://www.unrealengine.com/en-US/MetaHuman-creator.

14 https://www.virtualhumans.org/article/vlad-mastilovic-vp-of-digital-humans-technology-at-epic-games-tells-us-whats-next.

15 https://www.youtube.com/watch?v=qvnJ_wBiKUI&list=PLVnQ3VA8WbmJWeKdZ6iis0ikBNSKKol4S&index=5.

16 https://www.reallusion.com/character-creator/headshot/.

17 https://www.awn.com/animationworld/behind-scenes-final-fantasy-spirits-within.

18 Such comparison was possible thanks to the consultancy of the cosmetic surgeon Simona Centorbi.

19 https://3docean.net/item/low-poly-human-head-base-mesh/1582014.

20 Vertices are points in a 3d spaces, edges are connections between two vertices and faces are closed set of edges.

21 Example of texture: https://www.3dscanstore.com/male-multi-texture-base-mesh.

22 Example of UV mapping: https://en.wikipedia.org/wiki/UV_mapping#/media/File:UVMapping.png.

23 Example of topology: https://sketchfab.com/3d-models/human-head-topology-female-fcfb9195e1354017b93547f154aaee8b.

24 https://manual.reallusion.com/Character_Creator_3/ENU/3/Content/Character_Creator_3/3.3/15_SkinGen/Basic-Concepts/Skin-and-Makeup-Layers-Concepts.htm.

25 All the following quotes comes from the official website of unreal engine: https://docs.MetaHuman.unrealengine.com/en-US/MetaHumansUnrealEngine/MetaHumansLODs/ and https://docs.unrealengine.com/4.27/en-US/Resources/Showcases/MetaHumans/.

26 https://knowledge.autodesk.com/support/maya/learn-explore/caas/CloudHelp/cloudhelp/2017/ENU/Maya/files/GUID-89E3E944-01A3-452E-9518-D7F7D997DF8A-htm.html.

27 https://techcrunch.com/2021/02/10/epic-shows-off-unreals-nearly-real-MetaHuman-3d-character-creator/.

28 https://www.youtube.com/watch?v=OeUBATSJSr0.

29 https://manual.reallusion.com/Character_Creator_3/ENU/3/Content/Character_Creator_3/3.4/08_Animation/Facial-Animation/Applying-Facial-Expression-Templates.htm.

30 https://manual.reallusion.com/Character_Creator_3/ENU/3/Content/Character_Creator_3/3.4/08_Animation/Facial-Animation/Customize-Character-Facial-Expressions.htm.

31 https://www.unrealengine.com/en-US/blog/now-available-video-guide-on-using-the-MetaHuman-facial-rig.

32 *Assassin’s Creed Unity* (2014).

33 https://www.stocksy.com/2085913/abstract-portrait-painting-of-a-face; https://diamondbynumbers.com/products/pablo-picasso-surrealism-artwork-5d-diamond-painting/.

34 https://www.vrfocus.com/2021/04/create-MetaHumans-with-epic-games-early-access-cloud-based-app/.

35 https://www.nintendo.com/games/detail/arcade-archives-mario-bros-switch/.

36 A good example may be the introductive cutscene of the videogame “Final Fantasy VIII” which can be seen here: https://www.youtube.com/watch?v=XyBensMp_MA.

37 A method of recreating the effect of light on the complex geometry of the face.

38 There is however some recent debate on this claim, which we cannot discuss here. For further details see Haas and Schwarzkopf (2018).

39 Top 10 Movie Crying Scenes: https://www.youtube.com/watch?v=Cnv1hTHUhGY.

40 https://www.awn.com/animationworld/behind-scenes-final-fantasy-spirits-within.

41 https://digitalhumans.com/.

## References

[CR1] Arcagni S (2018). L’occhio della macchina.

[CR2] Balas B, Horski J (2012). You can take the eyes out of the doll, but. Perception.

[CR3] Balas B (2013). Biological sex determines whether faces look real. Vis Cogn.

[CR4] Balas B, Tonsager C (2014). Face animacy is not all in the Eyes: Evidence from contrast chimeras. Perception.

[CR5] Balas B, Pacella J (2015). Artificial faces are harder to remember. Comput Hum Behav.

[CR6] Balas B, Pacella B (2017). Trustworthiness perception is disrupted in artificial faces. Comput Hum Behav.

[CR7] Barthes R (1957). Mythologies.

[CR8] Blake-Turner C (2020). Fake news, relevant alternatives, and the degradation of our epistemic environment. Inquiry.

[CR9] Creeber G, Royston M (2009). Digital cultures: understanding new media.

[CR10] De Haas B, Schwarzkopf DS (2018). Feature–location effects in the Thatcher illusion. J Vision.

[CR11] Di Carlo JJ, Zoccolan D, Rust NC (2012). How does the brain solve visual object recognition?. Neuron.

[CR12] Dimberg U, Thunberg M, Elmehed K (2000). Unconscious facial reactions to emotional facial expressions. Psychol Sci.

[CR13] Dimberg U, Thunberg M, Elmehed K (2000). Unconscious facial reactions to emotional facial expressions. Psychol Sci.

[CR14] Dondero MG (2020). The language of images: the forms and the forces.

[CR15] Eco U (1968). La struttura assente.

[CR16] Eco U (1975). Trattato di semiotica generale.

[CR17] Eco U (1979). Lector in Fabula. La cooperazione interpretative nei testi narrativi.

[CR18] Eco U (1997/2016) Kant e l’ornitorinco. La Nave di Teseo, Milano

[CR19] Ekman P (1970). Universal facial expressions of emotion. Calif Mental Health Res Digest.

[CR20] Ekman P (1994). Strong evidence for universals in facial expressions: a reply to Russell's mistaken critique. Psychol Bull.

[CR21] Ekman P, Davidson RJ, Friesen WV (1990). The Duchenne smile: emotional expression and brain physiology: II. J Pers Soc Psychol.

[CR22] Eugeni R (2010). Semiotica dei Media Le forme dell’esperienza.

[CR23] Eugeni R, Catricalà V, Biggio F, Dos Santos V, Giuliana GT (2020). Technologically modified self-centred worlds modes of presence as effects of sense in virtual, augmented, mixed and extended reality. Meaning-making in extended reality.

[CR24] Evans AD, Bender J, Lee K (2016). Can parents detect 8- to 16-year-olds’ lies? Parental biases, confidence, and accuracy. J Exp Child Psychol.

[CR25] Gaspar MC, Bennett PJ, Sekuler AB (2008). The effects of face inversion and contrast-reversal on efficiency and internal noise. Vision Res.

[CR26] Greimas A-J, Courtès J (1979). Sémiotique - Dictionnaire raisonné de la théorie du langage.

[CR27] Geiger AR, Balas B (2021). Robot faces elicit responses intermediate to human faces and objects at face-sensitive ERP components. Sci Rep.

[CR28] Giuliana GT, Leone M (2021). Il volto nei giochi digitali: Funzioni e valori. Volti artificiali/artificial faces.

[CR29] Hartshorne C, Weiss P (1932). Collected papers of charles sanders peirce, volumes I and II: principles of philosophy and elements of logic.

[CR30] Iacoboni M (2008). I neuroni specchio.

[CR31] Korb S, Massaccesi C (2020). Angry men and happy women—a pre-registered replication using psychophysics. PsyArXiv.

[CR32] Korb S, Deniz TC, Ünal B (2021). Emotion perception bias associated with the hijab in Austrian and Turkish participants. Q J Exp Psychol (hove).

[CR33] Le Cun Y (2019). Quand la machine apprend. La révolution des neurones artificiels et de l’apprentissage profond.

[CR34] Lee-Morrison L (2019). Portraits of automated facial recognition. On machinic ways of seeing the face.

[CR35] Leone M (ed) (2021a) Volti artificiali/artificial faces. Special issue of Lexia. pp 37–38

[CR36] Leone M (2021b) Preface of Leone M (ed) Volti artificiali/artificial faces. Special issue of Lexia. Pp 37–38

[CR37] Leone M (2021c) Visage Mathematics: Semiotic Ideologies of Facial Measurement and Calculus. https://www.academia.edu/65641853/2021b_Visage_Mathematics_Semiotic_Ideologies_of_Facial_Measurement_and_Calculus

[CR38] Leone M (2021). Indexes: cultural nature and natural culture. Riv Estet.

[CR39] Leslie KR, Johnson-Frey SH, Grafton ST (2004). Functional imaging of face and hand imitation: towards a motor theory of empathy. Neuroimage.

[CR40] Levy P (1997). Il virtuale.

[CR41] Lotman JM (2006). Tesi per una semiotica delle culture.

[CR42] Lopatina OL, Komleva YK, Gorina YV (2018). Neurobiological aspects of face recognition: the role of oxytocin. Front Behav Neurosci.

[CR43] Malafouris L (2013). How things shape the mind.

[CR44] Magli P (2016). Il volto raccontato.

[CR45] Manovich L (2010). Software culture.

[CR46] Marini M, Ansani A, Paglieri F (2021). The impact of facemasks on emotion recognition, trust attribution and re-identification. Sci Rep.

[CR47] Masahiro M, MacDorman K, Norri K (2012). The uncanny valley [from the field]. IEEE Robot Autom Mag.

[CR48] Maurer D, Grand RL, Mondloch CJ (2002). The many faces of configural processing. Trends Cogn Sci.

[CR49] Meike R, Gobbini MI (2018). Familiarity matters: a review on prioritized processing of personally familiar faces. Vis Cogn.

[CR50] Monnet L (2004). A-life and the uncanny in “final fantasy: the spirits within”. Sci Fict Stud.

[CR51] Murphy J, Gray KL, Cook R (2017). The composite face illusion. Psychon Bull Rev.

[CR52] Niklas U, Pelc J, Sebeok TA, Stankiewicz E, Winner TG (2020). On the type—token distinction in C. S. Peirce. Sign, system and function: papers of the first and second polish-American Semiotics Colloquia.

[CR53] Palmer Colin J. (2020). Face Pareidolia Recruits Mechanisms for Detecting Human Social Attention. Psychological Science.

[CR54] Paolucci C (2010). Strutturalismo e Interpretazione.

[CR55] Paolucci C (2018). Three pragmatist legacies in the thought of Umberto Eco. Eur J Pragmat Am Philos.

[CR56] Paolucci C (2020). Persona. Soggettività nel linguaggio e semiotica dell’enunciazione.

[CR57] Paolucci C (2021). Cognitive semiotics: integrating signs, minds, meaning and cognition.

[CR58] Peirce CS (1992). The essential Peirce.

[CR59] Prieto L (1975). Pertinence et pratique.

[CR60] Prinz J (2006). Gut reactions: a perceptual theory of emotion.

[CR61] Rozenblatt D (2016) The Assassin's smile: facial expression as political expression. History of emotions—insights into research

[CR62] Russell JA (1995). Facial expressions of emotion: what lies beyond minimal universality?. Psychol Bull.

[CR63] Sadowski P, Bannink A, Honselaar W (2016). Between index and icon: Towards the semiotics of the cast shadow. (2016) From Variation to Iconicity: Festschrift for Olga Fischer.

[CR64] Schwind V, Wolf K, Henze N (2018). Avoiding the uncanny valley in virtual character design. Interactions.

[CR65] Stam R (2000). Film theory: an introduction.

[CR66] Sekuler AB, Gaspar CM, Gold JM, Bennett PJ (2004). Inversion leads to quantitative, not qualitative, changes in face processing. Curr Biol.

[CR67] Teufel C, Dakin SC, Fletcher PC (2018). Prior object-knowledge sharpens properties of early visual feature-detectors. Sci Rep.

[CR68] Thompson P (1980). Margaret thatcher: a new illusion. Perception.

[CR69] Todorov A (2017). Face value: the irresistible influence of first impressions.

[CR70] Tsao DY, Livingstone M (2008). Mechanism of face perception. Annu Rev Neurosci.

[CR71] Van Belle G, De Graef P, Verfaillie K, Rossion B, Lefèvre P (2010). Face inversion impairs holistic perception: evidence from gaze-contingent stimulation. J vis.

[CR72] Vecchio S (2020). Modi e questioni del credere. Rivista Italiana Di Filosofia Del Linguaggio.

[CR73] Viola M, Leone M (2021). Le espressioni facciali e i confini della semiotica. Volti artificiali/artificial faces.

[CR74] Volli U, Biggio F, Dos Santos V, Giuliana GT (2020). Archeologia semiotica del virtuale. (2020) Meaning-making in extended reality.

[CR75] Wetzel L (2018) Types and Tokens. In: Zalta ED (ed) The Stanford Encyclopedia of Philosophy (Fall 2018 Edition). https://plato.stanford.edu/archives/fall2018/entries/types-tokens

[CR76] Wetzel L (2018) "Types and Tokens", The Stanford Encyclopedia of Philosophy (Fall 2018 Edition), Edward N. Zalta (ed.)

[CR77] Yu N, Davis L and Fritz M (2019) Attributing fake images to GANs: learning and analyzing GAN fingerprints. Paper presented at 2019 IEEE/CVF International Conference on Computer Vision (ICCV), Seoul, Corea, Oct.27–Nov.2 2019

[CR78] Zhou G, Liu J, Xiao NG (2018). The fusiform face area plays a greater role in holistic processing for own-race faces than other-race faces. Front Hum Neurosci.

[CR79] Zikky M, Hariadi M, Muhtadin M (2013). Semi-automatic retargeting for facial expressions of 3D characters with fuzzy logic based on Blendshape interpolation. EMITTER Int J Eng Technol.

